# Cytomegalovirus infection associated with onset of ulcerative colitis

**DOI:** 10.1186/1756-0500-6-40

**Published:** 2013-02-02

**Authors:** Mitsuro Chiba, Toru Abe, Satoko Tsuda, Iwao Ono

**Affiliations:** 1 Division of Gastroenterology, Akita, Japan; 2 Department of Pathology, Nakadori General Hospital, Akita, Japan

**Keywords:** Cytomegalovirus, Ulcerative colitis, Cytomegalovirus colitis

## Abstract

**Background:**

In 2009, a trigger role of cytomegalovirus (CMV) was shown in the development of ulcerative colitis (UC) in mice. Fifteen cases of synchronous onset of CMV colitis and UC have been reported in literature. A careful prospective and retrospective survey identified CMV colitis in newly diagnosed UC patients at 4.5% (3/65 cases) and 8.2% (5/61 cases), respectively. This means that a majority of synchronous CMV colitis may be missed in newly diagnosed UC patients in routine practice. Such a case is presented.

**Case presentation:**

A 50-year-old woman, with a history of right partial mastectomy two years ago, had a persistent high fever for 9 days, after which a thickness of the colonic wall was detected on abdominal ultrasonography. Laboratory data showed inflammation and 2% atypical lymphocytes with the normal number of white blood cells. Although there was no bloody stool, fecal occult blood was over 1000 ng/ml. Colonoscopy showed diffuse inflammation in the entire large bowel and pseudomembranes in the sigmoid colon. The diagnosis was UC with antibiotic-associated pseudomembranous colitis. Metronidazole followed by sulfasalazine resulted in defervescence and improvement in laboratory data of inflammation. It took one month for normalization of fecal occult blood. Endoscopic remission was simultaneously confirmed. Later, it was found that a report of positive CMV antigenaemia (2/150,000) had been missed. Reevaluation of biopsy specimens using a monoclonal antibody against CMV identified positive cells, although inclusion bodies were not found in hematoxylin and eosin sections. Finally, the case was concluded to be synchronous onset of CMV colitis and UC.

**Conclusion:**

Synchronous CMV colitis is not routinely investigated in newly diagnosed UC patients. Together with a recent observation in animal studies, it is plausible that a subset (a few to several per cent) of UC patients develop synchronous CMV infection. Further studies are needed to elucidate the plausibility.

## Background

Cytomegalovirus (CMV) is a ubiquitous agent that asymptomatically infects the majority of persons [1,2]. Following infection, CMV remains lifelong in a latent state. Therefore, most cases of symptomatic CMV infection are caused by reactivation of a latent virus. CMV infection can occur in immunocompetent individuals, but it most frequently occurs in immunocompromised patients such as recipients of organ transplants, patients undergoing hemodialysis, patients receiving immunosuppressive drugs, and patients with acquired immune deficiency syndrome [2].

The association of CMV infection in severe cases of ulcerative colitis (UC) is well known and the CMV infection in these cases should be treated with an antiviral agent [3]. Advances in diagnostic techniques for CMV infection [4,5] have contributed to our understanding of UC associated with CMV. Domenech *et al.* prospectively studied the prevalence of CMV infection in five groups: active UC requiring intravenous steroids (n=25), steroid-refractory active UC treated with intravenous cyclosporine (n=19), inactive UC on azathioprine (n=25), inactive UC on mesalamine (n=25), and healthy controls (n=25) [6]. Only patients with steroid-refractory active UC (six of 19 patients, 32%) were compromised with CMV infection [6]. Using CMV antigenaemia assay and plasma polymerase chain reaction, it was found that reactivation of CMV up to 8 weeks after treatment with prednisolone and/or immunosuppressants such as cyclosporine was common (52.1%, 25/48) in moderate to severe UC, and CMV disappeared without antiviral therapy [7]. In the above cases, UC was diagnosed first, and CMV infection was identified later.

In 2009, a trigger role of CMV and norovirus was suggested in the development of UC and Crohn’s disease, respectively, in experimental murine systems [8-10]. Here, we report a case in which CMV colitis and UC synchronously developed.

## Case presentation

**Figure 1:**
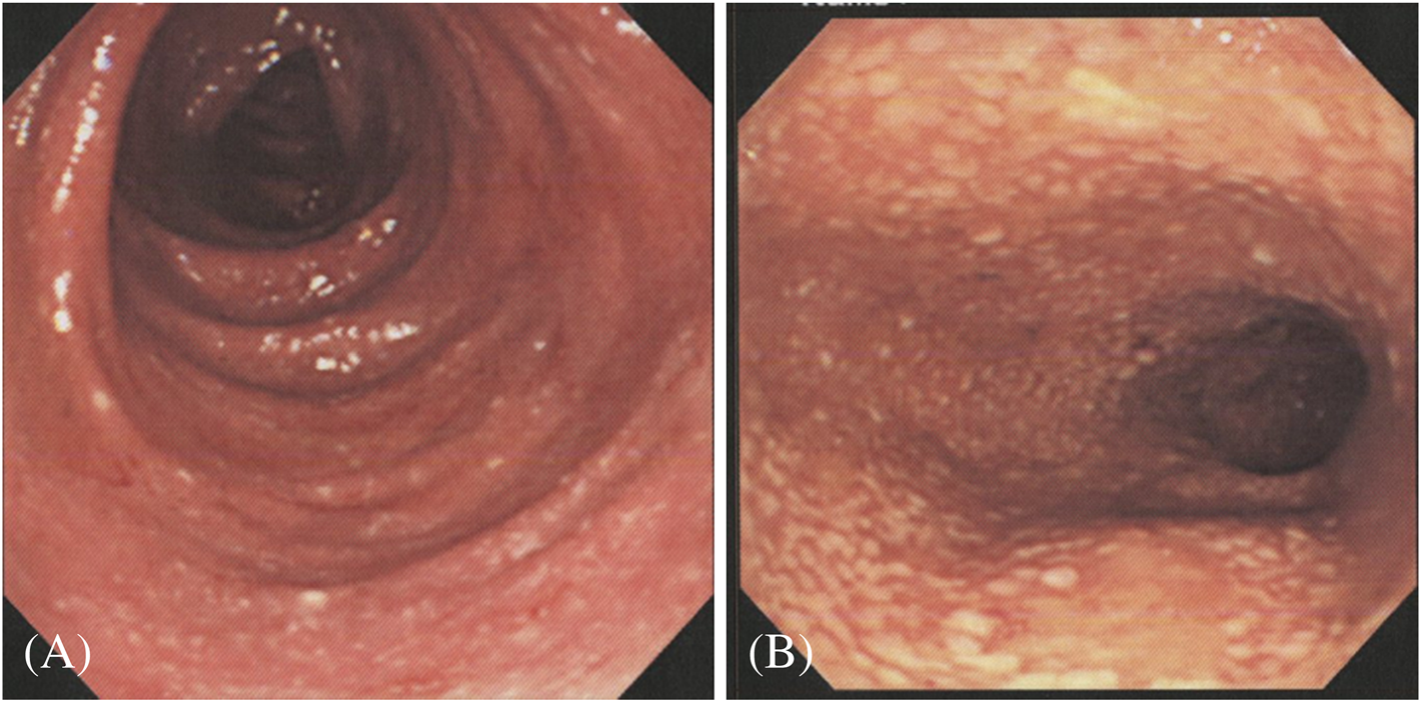
Diffuse inflammation in the ascending colon (A) and pseudomembranes in the sigmoid colon (B).

A 50-year-old woman underwent right partial mastectomy for breast cancer in December 2007, after which she was taking anastrozole (Arimidex^R^, AstraZeneca, Osaka, Japan), an aromatase inhibitor, as preventive therapy for breast cancer. There were no other relevant hospitalizations or regular medications. She experienced diarrhea for two days in late November 2009. Then a high fever above 38 centigrade persisted, so she visited the Outpatient Department for feverish patients (day 8). A laboratory test for influenza was negative. The following day, she visited the Division of Breast Surgery, where an antibiotic was prescribed for suspected urinary tract infection. Since the antibiotic was ineffective, she visited the Department of Internal Medicine (day 12). Abdominal ultrasonography revealed thickness of the bowel wall ranging from the transverse to the descending colon. She was referred and admitted to the Division of Gastroenterology (day 14). Laboratory data on admission showed elevated erythrocyte sedimentation rate (65 mm/hr: normal range, <10 mm/hr), high C-reactive protein (2.3 mg/dl: ≤0.3 mg/dl), elevated α_2_-globulin (11.5%: 4.8-8.6%), mild anemia (hemoglobin 11.5 g/dl: 12.0-15.1 g/dl), mild thrombocytosis (33.0 × 10^4^/mm^3^:15.2-31.4 × 10^4^/mm^3^), and 2% atypical lymphocytes with the normal number of white blood cells (7000/mm^3^: 3900-8800/mm^3^). Although there was no bloody stool, fecal occult blood was over 1000 ng/ml (normal range <100 ng/ml). Colonoscopy (day 15) showed diffuse inflammation without ulceration in the entire large bowel and pseudomembranes in the sigmoid colon (Figure 1A, B). The tentative diagnosis was UC with antibiotic-associated pseudomembranous colitis. Metronidazole 750 mg/day was started. *Clostridium difficile* toxin was negative and stool culture did not reveal any pathogen including enterohemorrhagic *E. coli*, *Campylobactor jejuni*, *Salmonella* species, *Staphylococcus aureus*, and *Krebsiella oxytoca*. Histology of the colon showed crypt abscesses consistent with UC. Therefore, a diagnosis of UC was made and sulfasalazine, 3 g/day, was started (day 22). The fever disappeared in a few days. Her laboratory data improved week by week. Fecal occult blood over 1000 ng/ml lasted until day 40. Fecal occult blood was negative on day 47. Colonoscopy on day 57 confirmed a morphological remission, and she was discharged on the following day.

**Figure 2:**
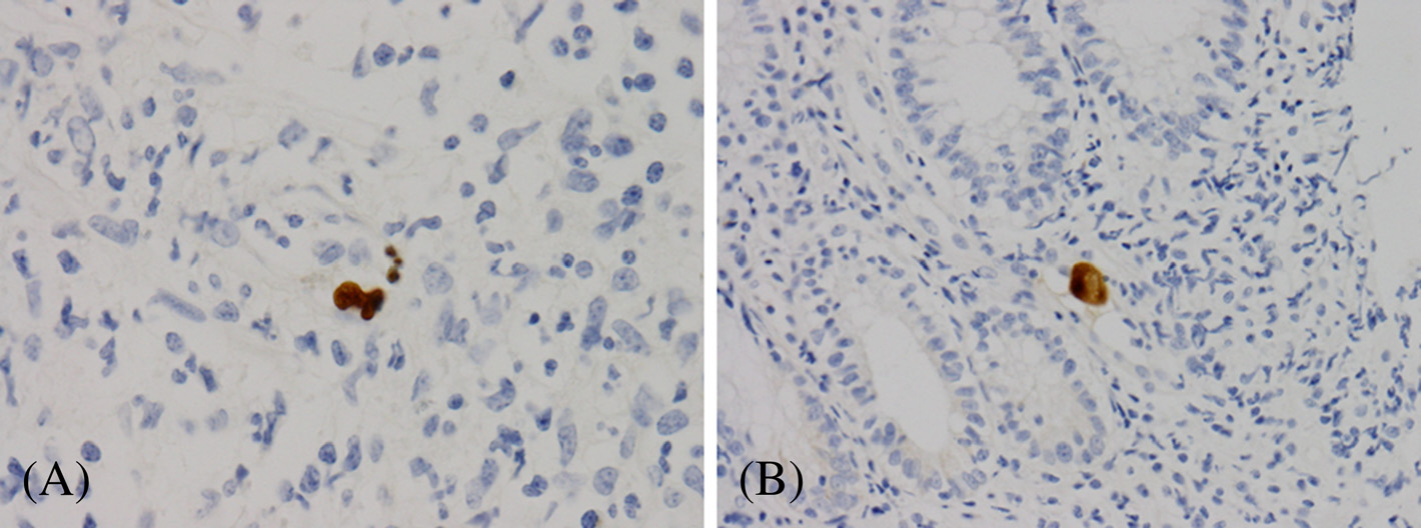
Positive cells in the ascending (x 400) (A) and sigmoid colon (x 200) (B) stained with monoclonal antibody against cytomegalovirus.

Reviewing her hospitalization, it was found that positive CMV antigenaemia (2/150,000 polymorphonuclear neutrophils: normal, no positive cells) tested on day 20 had been missed. Therefore, paraffin embedded colonic biopsy specimens were reevaluated immunohistochemically using a monoclonal antibody against CMV. Although inclusion bodies were not found in hematoxylin and eosin sections, immunohistologically positive cells were found in specimens from the ascending and sigmoid colon (Figure 2A, B). Disappearance of CMV antigenaemia and immunohistologically positive cells was ascertained on day 114 and day 125 respectively. She has been in remission to the present (September 2012).

## Discussion

It is difficult to explain this case as being solely CMV colitis. The most common endoscopic abnormality of CMV colitis is multiple ulcers [11,12]. When whole segments of colon are involved, lesions are skipped in CMV colitis [13]. In this case, ulcers were absent. The lesion was not skipped but continuous and showed crypt abscesses which are consistent with features of ulcerative colitis. In addition, clinical response was obtained with the drug for UC, sulfasalazine. Therefore, the present case can be concluded to be an association of CMV colitis and UC.

Initially this case was thought to be an atypical case of UC in which a high fever instead of bloody stool was manifested and pseudomembrane was observed in the sigmoid colon. Fever is one of the predominant features of CMV infection, and pseudomembrane has been reported in CMV colitis [14,15]. Therefore, these atypical phenomena can be explained by an involvement of CMV infection (colitis).

When UC developed the patient was taking anastrozole, aromatase inhibitor. Since hormone replacement therapy is a protective against relapse in IBD [16], the use of aromatase inhibitor which suppresses the production of estrogen might initiate IBD. However, no UC case associated with anastrozole has been reported to date. There is no UC case among 494 Japanese reports of adverse effects of anastrozole between February 2001, when the drug was started to be used in Japan, and December 2012 (Center of Medical Information, AstraZeneca, Osaka, Japan).

Synchronous onset of CMV colitis and UC was first reported in 1990 [17]. Since then at least fifteen cases have been reported by nine authors (Table 1) [18-25]. These cases were primary infections rather than reactivation of CMV. During a retrospective survey of the prevalence of CMV colitis in UC by immunohistochemistry, Kim *et al.* made an intriguing finding: identification of CMV colitis in 8.2% (5/61) of newly diagnosed UC patients [24]. None of the five cases had inclusion bodies on hematoxylin and eosin stain; consequently, none of them was diagnosed with CMV colitis at the time of diagnosis of UC. This means that a majority of synchronous CMV colitis is missed in newly diagnosed UC patients in routine practice. As in Kim *et al*’s case [24], involvement of CMV colitis in the present case had been missed during her hospitalization.

**Table 1:** Synchronous onset of cytomegalovirus colitis and ulcerative colitis

	**Age/**	**Chief**	**CMV**	**Atypical**	**Histological**	**Type of UC**
	**Sex**	**complaint**	**infection**	**lymphocyte**	**diagnosis of**	**by extent**
					**CMV colitis**	
Diepersloot *et al.*, 1990 [17]	39/f	F, HA, AP, BD	primary	+	IB, IHC, ISH	P
Lortholary *et al*. 1993 [18]	27/f	F, BD	primary	5%	IB	Lt
Orvar *et al.*, 1993 [19]	33/f	F, AP, BD, WL	primary	n.d.	IB, IHC	P
Mate del Tio *et al.*, 1996 [20]	64/m	BD, WL	primary	0	IB, IHC	Lt
Aoyagi *et al.*, 1998 [21]	41/f	n.d.	n.d.	n.d.	IB, IHC	EC
Hussein *et al.*, 2006 [22]	29/m	F, AP, WD	primary	43%	IB: negative	EC
Martin *et al.*, 2006 [23]	28/m	F, AP, HA, BD	primary	18%	IB	Lt
Kim *et al.*, 2010 [24]	19/m	n.d.	n.d.	n.d.	IHC	Lt
	33/m	n.d.	n.d.	n.d.	IHC	EC
	43/f	n.d.	n.d.	n.d.	IHC	EC
	57/m	n.d.	n.d.	n.d.	IHC	EC
	40/f	n.d.	n.d.	n.d.	IHC	Lt
Kim *et al.*, 2012 [25]	58/m	n.d.	n.d.	n.d.	IHC	EC
	39/m	n.d.	n.d.	n.d.	IHC	Lt
	68/m	n.d.	n.d.	n.d.	IB, IHC	Lt
Present case	50/f	F	primary	2%	IHC	EC

UC, ulcerative colitis; CMV, cytomegalovirus; f, female; m, male; F, fever; HA, headache; AP, abdominal pain.

BD, bloody diarrhea; WL, weight loss; WD, watery diarrhea; n.d., not described; IB, inclusion body.

IHC, immunohistochemistry; ISH, in situ hybridization; P, proctosigmoiditis; Lt, left-sided colitis, EC, entire colitis.

De novo inflammatory bowel disease is an increasingly recognized entity: de novo inflammatory bowel disease, a more common UC than Crohn’s disease, develops after solid organ transplantation [26]. The incidence of de novo IBD in the transplanted patients is estimated to be ten times the expected incidence of IBD in the general population [27]. The main risk factor of de novo IBD has been found to be CMV infection [27-30]. Onyeagocha *et al*. investigated the significance of CMV infection on the development of colitis in a murine system [8]. Murine CMV (MCMV) infection resulted in lasting elevation of antibodies to gut commensal bacteria that is observed in human IBD [10]. Colitis developed following a trigger (dextran sodium sulfate) in a far more severe form in MCMV-infected mice than in mice treated by the trigger alone. They concluded that (latent) CMV infection may predispose to developing IBD.

## Conclusion

We have reported a case in which CMV colitis and UC synchronously developed. It is plausible that a subset (a few to several per cent) of UC patients develop synchronous CMV infection. Further studies are needed to elucidate the plausibility.

## Consent

Written informed consent was obtained from the patient for publication of this case report and any accompanying images. A copy of the written consent is available for review by the Editor-in-Chief of this journal.

## Competing interests

The authors declare that they have no competing interests.

## Authors’ contributions

MC is a responsible doctor for the patient, undertook barium enema study, and wrote the report. ST performed colonoscopy. TA contributed to the acquisition of data. IO performed microscopic studies. All authors read and approved the final manuscript.
